# Electroacupuncture for Functional Constipation: A Multicenter, Randomized, Control Trial

**DOI:** 10.1155/2017/1428943

**Published:** 2017-01-31

**Authors:** Xiao Wu, Cuihong Zheng, Xiaohu Xu, Pei Ding, Fan Xiong, Man Tian, Ying Wang, Haoxu Dong, Mingmin Zhang, Wei Wang, Shabei Xu, Minjie Xie, Guangying Huang

**Affiliations:** ^1^Institute of Integrated Traditional Chinese and Western Medicine, Tongji Hospital, Tongji Medical College, Huazhong University of Science and Technology, 1095 Jiefang Avenue, Wuhan, Hubei 430030, China; ^2^Department of Integrated Traditional Chinese and Western Medicine, Tongji Hospital, Tongji Medical College, Huazhong University of Science and Technology, 1095 Jiefang Avenue, Wuhan, Hubei 430030, China; ^3^Department of Neurology, Tongji Hospital, Tongji Medical College, Huazhong University of Science and Technology, 1095 Jiefang Avenue, Wuhan, Hubei 430030, China

## Abstract

*Background and Aim*. To investigate the efficacy and safety of electroacupuncture (EA) with different current intensities for functional constipation (FC) and to assess whether the effects of EA with different current intensities are superior to the mosapride.* Methods*. Patients with FC were randomly divided into low current intensity group (LCI), high current intensity group (HCI), and mosapride group (MC). The primary outcome was three or more spontaneous bowel movements (SBMs) per week and an increase of one or more SBMs from baseline during at least 3 of the 4 weeks.* Results*. The primary outcome was reached by 53.45%, 66.15%, and 52.24% of the patients who received LCI, HCI, and mosapride, respectively. EA can significantly improve the weekly SBMs and stool consistency and reduce straining severity (*p* < 0.0001, all). HCI improved the quality of life better than mosapride (*p* < 0.05) and reduced the proportion of severe constipation more than LCI and mosapride (*p* < 0.05, both).* Conclusions*. EA is effective and safe at both current intensities for FC; therapeutic effects of LCI and HCI are not superior to mosapride. EA is superior to mosapride in improving patients' life quality and satisfaction level of treatment; EA has fewer adverse events than mosapride.

## 1. Introduction 

Functional constipation (FC) is a common type of functional gastrointestinal disorders (FGIDs) [1]. Generally, constipation is defined as infrequent bowel movements (BMs), typically fewer than 3 times per week; patients have a more extensive set of symptoms, including hard or lumpy stools, straining, and a sensation of incomplete rectal evacuation, abdominal discomfort, bloating, a sense of anorectal blockage during defecation, and the need for manual maneuvers during defecation [2]. Symptoms of FC are extremely common that can negatively affect the quality of patients' life [3, 4]. Because of its high prevalence rate [5] and the severe symptoms, this disorder represents a large economic burden to the health care system [3, 6, 7]. Even though there are multiple medications (including prescription laxatives, over-the-counter products, and fibre supplementation) for chronic constipation, the level of satisfaction with treatments is still poor [4, 8]. Therefore, many patients turn to choose complementary and alternative options from traditional Chinese medicine (TCM) for their constipation symptoms [9].

Acupuncture is a crucial part of TCM, with thousands of years' history. Because of its convenience, safety, and unique therapeutic effects, acupuncture has gained increasing popularity in Western countries [10]. Electroacupuncture (EA), a modified procedure with acupuncture and electrical current stimulation, has been widely used in recent years [11] due to the convenience of controlling and regulating parameters of current stimulation. Numerous studies have been performed to investigate that acupuncture or EA can alter gastrointestinal motility functions and benefit patients with functional gastrointestinal diseases [12, 13]. According to a review [14], the most popular acupoints for FC are ST25 (Tianshu), ST37 (Shangjuxu), BL25 (Dachangshu), ST36 (Zusanli), and TE6 (Zhigou). Various acupoint groups have been used for constipation: abdomen acupoints (e.g., ST25) plus crus acupoints (e.g., ST36, ST37) or forearm acupoints (e.g., TE6, LI11) are the most common combinations. Studies use ST37 (Shangjuxu) combined with LI11 (Quchi) for functional constipation patients are infrequent. Different stimulation frequencies are used in these studies, including 2 Hz/200 Hz [15], 2 Hz/100 Hz [16], 2 Hz/15 Hz [17, 18], 2 Hz/10 Hz [19], and 10 Hz/50 Hz [20]. The frequency of 2/50 Hz is rarely used. Trials conducted with low current intensity and high current intensity of EA for functional constipation are especially rare.

For these reasons, we designed a multicenter, randomized, parallel, controlled trial using acupoints LI11 plus ST37 with a frequency of 2/50 Hz for functional constipation. In this study, our aims were (i) to evaluate the efficacy and safety of EA with different current intensities for patients with FC and (ii) to assess whether the effects of EA with different current intensities are superior to the mosapride.

## 2. Patients and Methods

### 2.1. Study Design

The total study period was 9 weeks, including 1-week baseline, 4-week treatment, and 4-week follow-up. The trial was conducted in accordance with the protocol [21], which is available with the full text at http://trialsjournal.biomedcentral.com/. This study was approved by the Clinical Trial Ethics Committee of Tongji Medical College, Huazhong University of Science and Technology (approval number FWA00007304), and was conducted in accordance with the provisions of the Declaration of Helsinki and Good Clinical Practice guidelines. This study was registered on the Clinical Trials system (ClinicalTrials.gov ID: NCT01274793).

### 2.2. Patients

Patients with FC (the diagnostic criteria were based on Rome III Criteria [22]) were recruited from the following three hospitals from December 14, 2011 (first patient enrolled), to March 29, 2015 (last patient completed): Affiliated Tongji Hospital of Huazhong University of Science and Technology, Campus Hospital of Huazhong University of Science and Technology, and Affiliated Hubei Provincial Hospital of Hubei University of TCM.

#### 2.2.1. Inclusion Criteria

Patients were included if they met all of the following criteria: (i) diagnosis of functional constipation according to the Rome III Criteria; (ii) aged between 18 and 70 years old (the range of age initially was from 18 to 65 years; during the trial, many patients aged older than 65 years were strongly willing to participant in the trial, and they met all the other inclusion criteria. Participants in some trials [23–25] conducted for constipation were older than 80 years. The protocol was immediately amended in accordance with that recommendation, the age of participants were expanded to not older than 70 years); (iii) not taking any drugs that promote gastrointestinal movements at least during the 1 week prior to randomization; (iv) willing to sign the informed consent form before randomization.

#### 2.2.2. Exclusion Criteria

The exclusion criteria were as follows: (i) unconsciousness, psychosis, or failure to express subjective symptoms; (ii) being complicated with serious cardiovascular, hepatic, or renal diseases, or bleeding disorders; (iii) being combined with progressing malignancy or other serious debilitating illnesses; (iv) women in gestation or lactation periods.

Before randomization, all patients were (i) asked to discontinue any medications for constipation symptoms (e.g., anticholinergic agents, narcotics, and laxatives) (but glycerine enema up to 10 mL daily was allowed as a rescue medication if a patient did not have defecation for three or more consecutive days; the details about the use of rescue medication were recorded in patient diaries) and (ii) fully informed and asked to sign a written informed consent based on their own will.

During an initial screening period, all patients received routine tests of blood, urine, stool, and blood biochemical (ALT, AST, BUN, and Scr), electrocardiogram (ECG), and a colonoscopy prior to randomization. These tests would help identify and exclude patients who have serious heart, liver, kidney, or other severe diseases, and a colonoscopy would exclude organic diseases. To exclude pregnant women, urine HCG or blood *β*-HCG were tested for possible pregnancy. All the patients received routine tests of blood, urine, and stool after completing the treatment, which would help assess the adverse events.

All patients were required to record defecation diaries, including the times of spontaneous bowel movements (SBMs) per day, stool consistency, and severity of straining. The evaluation criteria of stool consistency was based on the Bristol Stool Form Scale (BSFS) [26], which ranges from 1 to 7, with lower scores indicating harder stool and higher scores indicating more liquid stool. Scores for straining severity range from 0 to 3, 0 indicating not at all, 1 a little bit, 2 a moderate amount, and 3 a great deal and an extreme amount. This course would be continuing to 8 weeks.

### 2.3. Randomization and Blinding

Patients were completely randomized into the low current intensity group (LCI), high current intensity group (HCI), and mosapride citrate tablet control group (MC) at a ratio of 1 : 1 : 1. We used R2.0 software to generate the randomization sequence. The designated researchers prepared the sequence through the use of sealed, opaque, sequentially numbered envelopes. One person at each hospital was responsible for the envelopes. To preserve masking, only the acupuncturists knew the treatment allocation. The patients and recruiters were all unaware of study-group assignments. Blinded evaluation (the curative effect was evaluated by a third party who did not know the assignment) and blinded statistical analysis were emphasized during the data collection and analysis stage.

Doctors from gastroenterology department of the local hospitals were invited to screen participants. All the licensed acupuncturists were experienced and had years of clinical training. And the investigators (recruiters, acupuncturists, and outcome assessors) processed unified training before participating in the research for the consistency of implementation.

### 2.4. Sample Size

Sample size was calculated based on a study [27]; the mean weekly rates of SBMs in FC patients were 2.6 times per week with a standard deviation of 2.2 after drug treatment. Moreover, there was a 1.4-fold difference in the clinical effects between the drug and the placebo. Consequently, the desired mean defecating frequency was 4 times with a standard deviation of 3 after EA treatment. With an *α* level of 0.05 and a power of 90% [28] to detect statistically significant difference, a sample size of 213 (71 in each group) was needed and expanded to 243 (81 for each group) in consideration of a drop-out proportion of 15%.

### 2.5. Interventions

Patients in the electroacupuncture groups received 16 sessions of acupuncture treatments: 5 times per week (once a day for 5 days continuously, followed by a 2-day interval) during the first 2 weeks and 3 times per week (once every 2-3 days) during the following 2 weeks. Each session lasted 30 min.

LI11 and ST37 are the common used acupoints for functional gastrointestinal motility disorders [14, 17, 29–31]. In this study, acupoints of bilateral LI11 (Quchi, located at the midpoint between the lateral end of the transverse cubical crease and the lateral epicondyle of the humerus) and ST37 (Shangjuxu, located 6 cun below the lateral depression between the patellar and patellar ligament, one finger width lateral to the anterior crest of the tibia) were used.

After sterilizing the skin, acupuncture needles (0.30 × 40 mm or 0.30 × 50 mm, Human Health, Shanghai, China) were inserted into LI11 and ST37 for 15–25 mm vertically and slowly;* De qi* sensation (soreness, numbness, distension, and heaviness) was achieved through lifting and thrusting movements combined with twirling the needles. Then, auxiliary needles (0.18 × 13 mm, Human Health, Shanghai, China) were inserted into the proximal limbs with 2 mm lateral to the first needle for 5 mm vertically, without manual stimulation. The acupuncture needle and auxiliary needle of each point were connected with an electroacupuncture instrument (HANS-200E, Nanjing, Jisheng, Jiangsu, China) to form a circuit that lasted for 30 min, with a dilatational wave at a frequency of 2/50 Hz. For the LCI group, the current applied was relatively weak but can be clearly perceived by the participants. For the HCI group, the current was strong enough to reach the patients' tolerance threshold value.

Patients in mosapride control group were orally given 5 mg mosapride citrate tablet (Dainippon Sumitomo pharmaceutical Co. Ltd., Japan) 3 times daily for 4 continuous weeks if no severe adverse events were detected.

### 2.6. Assessments

The primary outcome was defined as both three or more SBMs per week and an increase of one or more SBMs per week from baseline for 3 or more weeks during 4-week treatment period [25]. Secondary outcomes included the change from baseline of mean stool frequency (weekly rates of SBMs from week 1 to week 8), stool consistency, and severity of straining during the 9 weeks of the study. A number of additional outcomes were assessed, including the proportion of patients who belong to severe constipation (defined as weekly SBMs less than 2 times per week [27]), the strength of association between baseline values and the presence of primary outcome and weekly SBMs ≥3 dichotomized as present/absent, and the validated Patient Assessment of Constipation Quality of Life (PAC-QOL) [32]. The PAC-QOL was assessed at baseline, weeks 2, 4, and 8, with lower scores indicating a better quality of life. Adverse events were also assessed.

### 2.7. Statistical Analysis

SAS statistical package program (ver. 9.2, SAS Institute, Cary NC, USA) was used. All *p* values were based on two-sided tests; *p* < 0.05 was considered to be a statistically significant difference. Statistical analysis of our crowd included full analysis set (FAS, the main set of therapeutic evaluation and analysis) and safety set (SS, the main set of safety evaluation). Efficacy analysis was based on an intent-to-treat population. Continuous variables were presented as mean ± SD (standard deviation) or mean (95% confidence interval [CI]); categorical variables were expressed by frequency and percentage unless stated otherwise.

Categorical variables were analyzed with the used of the Cochran–Mantel–Haenszel-*χ*^2^ test (CMH-*χ*^2^). Continuous variables comparison of baseline period among the three groups was analyzed with the analysis of variance (ANOVA). And an analysis of covariance (ANCOVA) with fixed-effect terms for study group and center and with the corresponding baseline value as a covariate was used for the comparison of treatment and follow-up periods among the three groups. Finally, we used least significant difference (LSD) for further pairwise comparison if there was statistic significance of difference.

Logistic regression analysis was used to estimate the strength of association between belonging to severe constipation and the presence of primary outcome and weekly SBMs ≥3 dichotomized as present/absent. The total number of SBMs was summed and divided by 4 (the number of weeks of treatment and follow-up). The data were also summarized for each of the two 2-week periods of the 4-week treatment study. Two models were examined for two different independent variables: model 1 adjusted for primary outcome and model 2 adjusted for weekly SBMs ≥3. Age, sex, body mass index (BMI), severe constipation, duration of constipation, group status, occupation, and education were included as covariables. We also calculated odds ratios (ORs) and 95% confidence intervals (CIs).

Patients were assumed not to have had bowel movements or to have taken rescue medications if the corresponding daily question was not answered. We used last observation carried forward (LOCF) for the missing data of the primary outcome and the secondary outcomes.

## 3. Results

### 3.1. Outcomes

**Figure 1:**
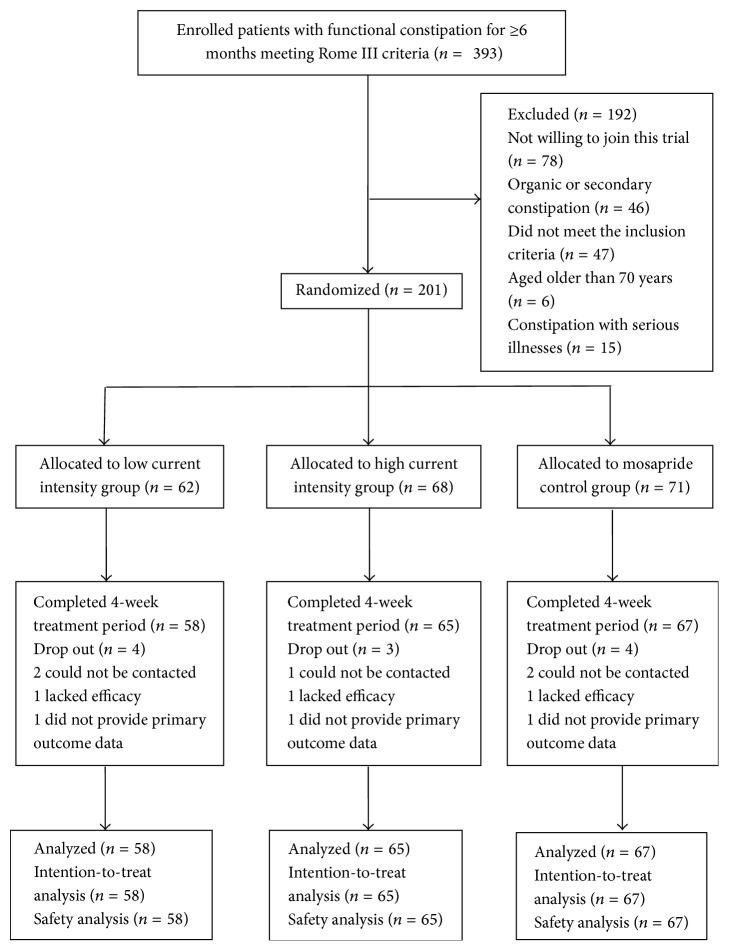
Flow chart of study participants.

Of the 201 patients who signed consent forms, 190 were randomly assigned to the three groups and received respective therapies (more information is detailed in Figure 1). Baseline demographic and clinical characteristics of patients in the three groups are detailed in Table 1.

#### 3.1.1. Primary Outcome

**Figure 2:**
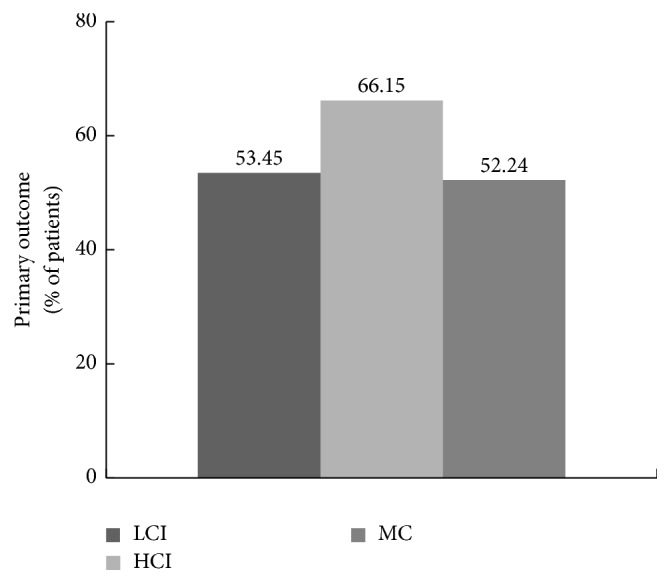
The primary outcome in the LCI, HCI, and mosapride groups. The primary outcome was defined as a weekly frequency of three or more spontaneous bowel movements (SBMs) and an increase of one or more SBMs from baseline for at least 3 weeks of the 4-week treatment period. The Cochran–Mantel–Haenszel-*χ*^2^ (CMH-*χ*^2^) test was used.

Among the three groups, respectively, 53.45%, 66.15%, and 52.24% of the patients in LCI, HCI, and mosapride groups reached the primary outcome (*p* > 0.05, among the three groups) (Figure 2).

#### 3.1.2. Secondary Outcomes

**Figure 3:**
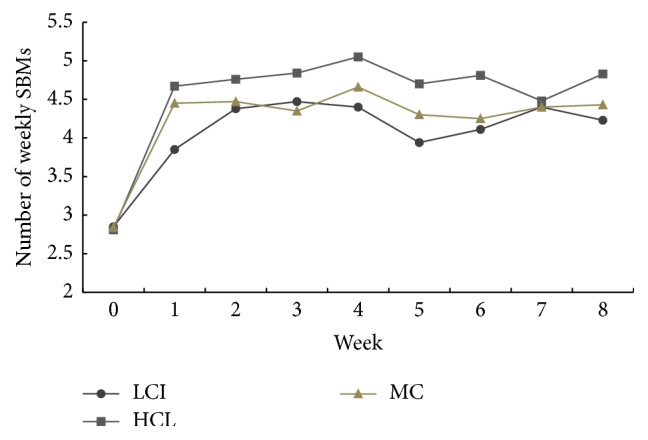
Mean number of weekly spontaneous bowel movements. The LCI, HCI, and mosapride resulted in a significant increase in the number of weekly SBMs, as compared with baseline period at each time frame from week 1 to week 8, respectively (*p* < 0.0001, all, while in LCI, at week 5 and week 8, *p* = 0.001, and *p* = 0.0001, resp.). Signed-Rank test was used.

The EA groups and MC group had significant improvements compared with baseline period, including the mean SBMs/week from week 1 to week 8 (Figure 3), the stool consistency, and severity of straining at weeks 2, 4, and 8 (Table 2).

#### 3.1.3. Additional Outcomes

The EA groups and MC group both reduced the proportion of severe constipation compared with baseline at weeks 2, 4, and 8, respectively. Moreover, at week 8, the proportion of severe constipation in HCL was remarkably less than LCI and MC (*p* < 0.05, both) (Table 3).

More patients who belong to severe constipation fulfilled the outcomes than those who were not belonging to severe constipation, including the primary outcome and the weekly SBMs ≥3 in the EA groups and mosapride (*p* < 0.05, all), except in mosapride group, with adjusted OR 1.42 and 95% CI 0.45–4.47 when analyzing the primary outcome (Table 4). In PAC-QOL, the EA groups and MC group had significant improvements on all subscales at weeks 2, 4, and 8, respectively, compared with baseline period (*p* < 0.0001, all) (Table 5).

### 3.2. Adverse Events

The total proportion of adverse events was 2.11% (4/190) in our trial. No adverse events were found in the LCI and HCI groups. In the mosapride group, 1 patient (1.49%, 1/67) reported diarrhea, 2 patients (2.99%, 2/67) experienced stomachache, and 1 patient (1.49%, 1/67) had upper respiratory infection. The difference was significant between the electroacupuncture groups and mosapride (*p* = 0.0143, among the three groups).

## 4. Discussion

The results of the 9-week multicenter, randomized, controlled trial show that all the LCI, HCI, and MC groups significantly increased the proportion of patients who fulfilled the primary outcome of three or more SBMs per week, with an increase from baseline of at least one SBM per week for 3 or more weeks of the 4-week treatment period. The outcome is rigorous, requiring a normal bowel function of patients (since ≥3 weekly SBMs are considered the low end of the range that defines normal bowel function [27]) and a sustainability of at least 75% of the treatment period [25]. Weekly SBMs are an important characteristic of bowel function, because reduced defecation frequency is typical for functional constipation [33]. The relief of functional constipation can be accurately and objectively reflected through the change of weekly SBMs [18]. Although this primary outcome was rigor, 66.15% of the patients who received high current intensity treatment still fulfilled the outcome, while 53.45% and 52.24% patients, respectively, in the LCI and MC were considered to have had a response.

Mosapride is a 5-HT_4_ receptor agonist and can stimulate upper gastrointestinal movement [34], accelerate gastric emptying [35] and colonic motility [36, 37], and improve stool frequency and consistency, especially in patients with irritable bowel syndrome with constipation (IBS-C) [38] and/or functional constipation [37]. Consequently, the effects of EA during the treatment and follow-up periods can be more accurately revealed by using mosapride as the controlled intervention.

Besides improving weekly SBMs, the EA groups significantly improved stool consistency and reduced straining severity, which is in accordance with previous studies [14, 17, 18, 39, 40]. And the effects of EA on symptoms of constipation were sustained through 8 weeks, including the treatment and follow-up periods. The secondary outcomes supported the findings of the primary outcome.

The LCI and HCI significantly reduced the proportion of severe constipation patients; this is consistent with a previous study [41]. Patients with severe constipation are associated with a significantly greater risk of developing colorectal cancer and benign colorectal neoplasms than constipation-free patients [42]. Therefore, the more than 50% reduction was considered to be clinically meaningful. Moreover, at week 8, proportion of severe patients in HCI less than LCI and MC was detected; EA treatment was superior to that of mosapride at follow-up period, suggesting that the EA treatment had better sustained effects than mosapride. Effects of acupuncture for constipation could last for 24 weeks [43], but pharmaceuticals, such as osmotic laxatives [18] and prokinetic agents [44], are generally thought to lack sustained effects.

Severe constipation patients can better improve the weekly SBMs than those patients who had no severe constipation. This is similar to a previous study [24] that medication treatment can better improve the baseline abdominal symptoms in the severe subpopulations than that population, which included patients with milder baseline abdominal symptoms. The significant difference presented rising trend in HCI, while exhibiting declining trend in mosapride, which is in accordance with that fewer proportion of severe constipation patients in HCI than LCI and mosapride at follow-up period.

The EA treatment remarkably improved the disease-related quality of patients' life, which is consistent with a previous study [45]. The PAC-QOL is a useful constipation patient-reported outcomes assessment tool, measuring patient health-related quality of life and satisfaction [32]. Improvements in PAC-QOL overall score and satisfaction score are associated with improvements in symptoms of chronic constipation [46]. Our data suggested that HCI group showed greater improvement than mosapride on satisfaction and worries and concerns subscales; moreover, HCI was better than LCI in improving level of satisfaction at week 4 and worries and concerns condition at week 8. It indicates that EA treatment is superior to that of mosapride in improving the patients' life quality and the satisfaction level of treatment.

In our trial, the total proportion of adverse events was 2.11%, only 4 patients from the mosapride group had adverse events, and no adverse events were found in the LCI and HCI groups. EA or acupuncture had no serious adverse events reported in treatment of FC [17] or IBS [47]. Acupuncture had less emergency drug usage and side effects than lactulose in the treatment of FC [18]. EA treatment is safer than mosapride for FC. The occurrence of adverse events in mosapride is not surprising, since mosapride is well tolerated, with diarrhea, loose stools, dry mouth, malaise, and headache being reported in <5% of patients [48]. Thus, the patients who are not willing to take pharmacologic treatment and/or have contraindications to agents can choose acupuncture for their constipation symptoms.

Our data suggest that EA treatment is effective and safe at both current intensities in the improvement of functional constipation. There is no significant difference between LCI and HCI in intestinal function except for proportion of severe constipation patients at week 8. A previous study [18] also indicated that deep needing and shallow needing both improve the symptoms of FC. It has long been known that acupuncture is an individualized treatment rather than a standardized needle manipulation [49]; the defined low and high intensity in the current study are according to the patients experience. Moreover, in the LCI and HCI, all patients achieved the* De qi *sensation;* De qi* is the sine qua non of acupuncture for the achievement of a clinical therapeutic effect according to TCM [50]. And EA stimulation can strengthen the* De qi* sensation. However, sufficient evidence with large sample size and long treatment and follow-up period studies to prove the connection of the current intensity parameter and the therapeutic effects of EA is needed.

The limitations of this clinical trial should be addressed. First, only different current intensities of EA were compared, no placebo control or no sham acupuncture control; it might not be accurate enough to reflect the therapeutic effects of EA. An inadequate number of patients to prove the efficacy were another limitation; possible reasons are as follows: (i) we had only three hospitals to help us recruit FC patients; (ii) some patients with lighter symptoms who did not care about their problems might pay little attentions to the information from our hospitals; (iii) some other patients had insufficient time to participate in our clinical trial. Besides, our 1-week screening period is slightly short, and our 4-week treatment period and 4-week follow-up period might not be long enough for the interventions to show completely the effects of EA. Patients might have taken laxatives or stool softeners before study enrollment; the 1-week screening period might not accurately reflect the severity of constipation patients. Acupuncture had better sustained effects than pharmaceuticals [18]; therefore, the follow-up period should be long enough that can accurately reflect the sustained effects of acupuncture. More rigorous and high-quality studies with larger sample sizes are required.

In conclusion, the EA treatment is effective and safe at both current intensities for patients with functional constipation. And therapeutic effects of low and high current intensity are not superior to that of mosapride in improving the weekly SBMs, stool consistency, and straining severity. However, EA treatment is superior to that of mosapride in improving patients' life quality and satisfaction level of treatment. Meanwhile, EA treatment has fewer adverse events compared with mosapride. These findings might qualify the superiority of EA.

## Figures and Tables

**Table 1:** Demographic and baseline characteristics of the patients (intention-to-treat population).

Characteristic	LCI (*n* = 58)	HCI (*n* = 65)	MC (*n* = 67)	*p* value
Sex, *n* (%)				
Female	52 (89.66)	60 (92.31)	57 (85.07)	0.41
Age, years, mean ± SD	34.00 ± 15.62	37.20 ± 18.19	43.60 ± 17.90	0.55
Range	20.00–63.00	22.00–62.00	23.00–69.00	
BMI, mean ± SD	20.95 ± 2.36	21.22 ± 2.98	21.10 ± 2.18	0.83
Education, *n* (%)				0.09
Postgraduate	8 (13.79)	15 (23.08)	21 (31.34)	
Bachelor	31 (53.45)	26 (40.00)	21 (31.34)	
Junior college	4 (6.90)	9 (13.85)	7 (10.45)	
Senior middle school	15 (25.86)	13 (20.00)	15 (22.39)	
Junior middle school	0 (0.00)	2 (3.08)	3 (4.48)	
Duration of constipation, months, mean ± SD	70.44 ± 85.53	86.29 ± 104.06	68.09 ± 74.13	0.45
Severe constipation, *n* (%)	34 (58.62)	32 (49.23)	34 (50.75)	0.54

The analysis of variance (ANOVA) for continuous variables and the Cochran–Mantel–Haenszel-*χ*^2^ (CMH-*χ*^2^) test for categorical variables. *p* values of the comparison among the three groups. Severe constipation defined as spontaneous bowel movements less than 2 times per week.

**Table 2:** Secondary outcomes.

	Mean (95% CI)			
	LCI (*n* = 58)	HCI (*n* = 65)	MC (*n* = 67)	*p* value
Weekly SBMs				
Baseline	2.85 (2.32, 3.38)	2.81 (2.37, 3.25)	2.84 (2.38, 3.30)	0.98
Week 2 change from baseline	1.53 (1.03, 2.03)^*∗∗∗*^	1.95 (1.49, 2.41)^*∗∗∗*^	1.63 (1.17, 2.09)^*∗∗∗*^	0.58
Week 4 change from baseline	1.55 (0.93, 2.17)^*∗∗∗*^	2.24 (1.81, 2.67)^*∗∗∗*^	1.81 (1.37, 2.25)^*∗∗∗*^	0.21
Week 8 change from baseline	1.38 (0.74, 2.02)^*∗∗*^	2.02 (1.62, 2.42)^*∗∗∗*^	1.62 (1.14, 2.10)^*∗∗∗*^	0.19
Stool consistency				
Baseline	2.40 (2.12, 2.68)	2.46 (2.21, 2.71)	2.28 (2.01, 2.55)	0.57
Week 2 change from baseline	0.64 (0.38, 0.90)^*∗∗∗*^	0.97 (0.66, 1.28)^*∗∗∗*^	0.92 (0.66, 1.18)^*∗∗∗*^	0.27
Week 4 change from baseline	0.78 (0.45, 1.11)^*∗∗∗*^	0.80 (0.58, 1.02)^*∗∗∗*^	0.95 (0.64, 1.26)^*∗∗∗*^	0.93
Week 8 change from baseline	0.99 (0.73, 1.25)^*∗∗∗*^	0.79 (0.53, 1.05)^*∗∗∗*^	0.85 (0.57, 1.13)^*∗∗∗*^	0.73
Straining severity				
Baseline	1.20 (1.04, 1.36)	1.33 (1.19, 1.47)	1.23 (1.07, 1.39)	0.54
Week 2 change from baseline	−0.50 (−0.67, −0.33)^*∗∗∗*^	−0.49 (−0.65, −0.33)^*∗∗∗*^	−0.33 (−0.49, −0.17)^*∗∗*^	0.14
Week 4 change from baseline	−0.44 (−0.62, −0.26)^*∗∗∗*^	−0.53 (−0.71, −0.35)^*∗∗∗*^	−0.41 (−0.60, −0.22)^*∗∗*^	0.38
Week 8 change from baseline	−0.54 (−0.77, −0.31)^*∗∗∗*^	−0.59 (−0.76, −0.42)^*∗∗∗*^	−0.34 (−0.52, −0.16)^*∗∗*^	0.20

^*∗∗∗*^ *p* < 0.0001 versus baseline; ^*∗∗*^*p* < 0.001 versus baseline.

^*∗∗∗*^ *p* and ^*∗∗*^*p* values were used through the Signed-Rank test.

*p* values were for the comparison among the three groups and were calculated with the analysis of covariance (ANCOVA), except for the comparison of the baseline values, which used an analysis of variance (ANOVA).

(1) SBMs denote spontaneous bowel movements.

(2) Stool consistency was assessed with the use of the 7-point Bristol Stool Form Scale (BSFS): 1 indicating separate, hard lumps, like nuts (hard to pass); 2 sausage-shaped but lumpy; 3 like a sausage but with cracks on the surface; 4 like a sausage or snake, smooth, and soft; 5 soft blobs with clear-cut edges (passed easily); 6 fluffy pieces with ragged edges or a mushy stool; and 7 watery, not solid pieces (entirely liquid).

(3) Straining severity was assessed by means of a 4-point ordinal scale with the following responses, while 0 indicates not at all, 1 a little bit, 2 a moderate amount, and 3 a great deal and an extreme amount.

**Table 3:** The proportion of severe constipation patients in LCI, HCI, and mosapride groups.

Severe constipation, *n* (%)	LCI (*n* = 58)	HCI (*n* = 65)	MC (*n* = 67)	*p* value
Baseline	34 (58.62)	32 (49.23)	34 (50.75)	0.54
2 W	20 (34.48)^*∗*^	12 (18.46)^*∗∗*^	17 (25.37)^*∗*^	0.13
4 W	15 (25.86)^*∗∗*^	12 (18.46)^*∗∗*^	17 (25.37)^*∗*^	0.54
8 W	15 (25.86)^*∗∗*^	6 (9.23)^*∗∗*¶#^	17 (25.37)^*∗*^	0.03

^*∗∗*^ *p* < 0.001, versus baseline; ^*∗*^*p* < 0.01, versus baseline. ^¶^*p* < 0.05, versus LCI; ^#^*p* < 0.05, versus mosapride.

Severe constipation defined as spontaneous bowel movements less than 2 times per week.

^*∗*^ *p* and ^*∗∗*^*p* values were used through the Signed-Rank test.

^#^ *p* and ^¶^*p* values were used through the least significant difference (LSD).

*p* values were for the comparison among the three groups and were calculated with the analysis of covariance (ANCOVA), except for the comparison of the baseline values, which used an analysis of variance (ANOVA).

**Table 4:** Primary outcome of and weekly SBMs ≥3 among patients belonging to severe constipation adjusted for baseline data.

	Adjusted OR (95% CI)		
	LCI (*n* = 58)	HCI (*n* = 65)	MC (*n* = 67)
Primary outcome	3.48 (1.06, 11.38)^*∗*^	3.67 (1.01, 13.27)^*∗*^	1.42 (0.45, 4.47)
Weekly SBMs ≥3			
Weeks 1-2	21.13 (3.26, 137.13)^*∗∗*^	12.15 (2.18, 67.61)^*∗∗*^	13.89 (2.51, 76.81)^*∗∗*^
Weeks 3-4	30.97 (3.31, 289.53)^*∗∗*^	17.51 (2.40, 127.69)^*∗∗*^	8.81 (2.17, 35.77)^*∗∗*^
Weeks 1–4	29.29 (3.54, 242.46)^*∗∗*^	29.31 (3.06, 281.29)^*∗∗*^	7.55 (1.76, 32.40)^*∗∗*^
Weeks 5–8	29.60 (3.85, 227.92)^*∗∗*^	40.30 (3.55, 457.07)^*∗∗*^	4.04 (1.15, 14.16)^*∗*^

CI = confidence interval; OR = odds ratio; ^*∗*^*p* < 0.05; ^*∗∗*^*p* < 0.01.

Severe constipation defined as spontaneous bowel movements less than 2 times per week.

Variables included in the model: age, sex, body mass index (BMI), severe constipation, duration of constipation, group status, occupation, and education.

**Table 5:** PAC-QOL questionnaire.

	Mean (95% CI)			
	LCI (*n* = 58)	HCI (*n* = 65)	MC (*n* = 67)	*p* value
Overall scores				
Week 2 change from baseline	0.67 (0.55, 0.79)^*∗∗∗*§^	0.63 (0.49, 0.77)^*∗∗∗*#^	0.40 (0.26, 0.54)^*∗∗∗*^	0.00
Week 4 change from baseline	0.72 (0.59, 0.85)^*∗∗∗*^	0.82 (0.68, 0.96)^*∗∗∗*#^	0.47 (0.35, 0.59)^*∗∗∗*^	0.00
Week 8 change from baseline	0.78 (0.64, 0.92)^*∗∗∗*^	0.93 (0.77, 1.09)^*∗∗∗*#^	0.56 (0.42, 0.70)^*∗∗∗*^	0.00
Physical discomfort				
Week 2 change from baseline	0.74 (0.56, 0.92)^*∗∗∗*^	0.67 (0.49, 0.85)^*∗∗∗*^	0.53 (0.34, 0.72)^*∗∗∗*^	0.26
Week 4 change from baseline	0.88 (0.72, 1.04)^*∗∗∗*^	0.92 (0.74, 1.10)^*∗∗∗*#^	0.56 (0.39, 0.73)^*∗∗∗*^	0.01
Week 8 change from baseline	0.91 (0.72, 1.10)^*∗∗∗*^	1.08 (0.90, 1.26)^*∗∗∗*^	0.72 (0.53, 0.91)^*∗∗∗*^	0.06
Psychosocial discomfort				
Week 2 change from baseline	0.44 (0.31, 0.57)^*∗∗∗*^	0.34 (0.23, 0.45)^*∗∗∗*^	0.27 (0.12, 0.42)^*∗∗∗*^	0.12
Week 4 change from baseline	0.47 (0.33, 0.61)^*∗∗∗*^	0.49 (0.33, 0.65)^*∗∗∗*^	0.32 (0.18, 0.46)^*∗∗∗*^	0.10
Week 8 change from baseline	0.57 (0.43, 0.71)^*∗∗∗*^	0.63 (0.43, 0.83)^*∗∗∗*^	0.44 (0.28, 0.60)^*∗∗∗*^	0.22
Worries and concerns				
Week 2 change from baseline	0.71 (0.57, 0.85)^*∗∗∗*§^	0.63 (0.46, 0.80)^*∗∗∗*#^	0.42 (0.26, 0.58)^*∗∗∗*^	0.02
Week 4 change from baseline	0.75 (0.59, 0.91)^*∗∗∗*^	0.86 (0.69, 1.03)^*∗∗∗*#^	0.52 (0.37, 0.67)^*∗∗∗*^	0.01
Week 8 change from baseline	0.79 (0.60, 0.98)^*∗∗∗*^	0.99 (0.81, 1.17)^*∗∗∗*#¶^	0.60 (0.43, 0.77)^*∗∗∗*^	0.01
Satisfaction				
Week 2 change from baseline	0.92 (0.67, 1.17)^*∗∗∗*^	1.02 (0.78, 1.26)^*∗∗∗*#^	0.45 (0.26, 0.64)^*∗∗∗*^	0.01
Week 4 change from baseline	0.93 (0.69, 1.17)^*∗∗∗*^	1.15 (0.89, 1.41)^*∗∗∗*#¶^	0.52 (0.33, 0.71)^*∗∗∗*^	0.00
Week 8 change from baseline	1.00 (0.78, 1.22)^*∗∗∗*^	1.18 (0.91, 1.45)^*∗∗∗*#^	0.54 (0.30, 0.78)^*∗∗∗*^	0.00

^*∗∗∗*^ *p* < 0.0001, versus baseline; ^#^*p* < 0.05, versus mosapride.

^§^ *p* < 0.05, versus mosapride; ^¶^*p* < 0.05, versus LCI

^*∗∗∗*^ *p* values were used through the Signed-Rank test.

^#^ *p*, ^¶^*p*, and ^§^*p* values were used through the least significant difference (LSD).

*p* values were for the comparison among the three groups and were calculated with the analysis of covariance (ANCOVA), except for the comparison of the baseline values, which used an analysis of variance (ANOVA).
